# IT Infrastructure to Support the Secondary Use of Routinely Acquired Clinical Imaging Data for Research

**DOI:** 10.1007/s12021-014-9240-7

**Published:** 2014-08-17

**Authors:** Kai Yan Eugene Leung, Fedde van der Lijn, Henri A. Vrooman, Miriam C. J. M. Sturkenboom, Wiro J. Niessen

**Affiliations:** 1 Department of Medical Informatics, Erasmus MC: University Medical Center Rotterdam, Dr. Molewaterplein 50, Building NA, Room NA2502, 3015 GE Rotterdam, Zuid-Holland The Netherlands; 2 Department of Radiology, Erasmus MC: University Medical Center Rotterdam, Rotterdam, The Netherlands; 3 Department of Epidemiology, Erasmus MC: University Medical Center Rotterdam, Rotterdam, The Netherlands; 4 Faculty of Applied Sciences, Department of Imaging Science & Technology, Quantitative Imaging Group, Delft University of Technology, Delft, The Netherlands

**Keywords:** Medical informatics, Magnetic resonance imaging, Radiology information systems, Neuroimaging, Biomarkers

## Abstract

We propose an infrastructure for the automated anonymization, extraction and processing of image data stored in clinical data repositories to make routinely acquired imaging data available for research purposes. The automated system, which was tested in the context of analyzing routinely acquired MR brain imaging data, consists of four modules: subject selection using PACS query, anonymization of privacy sensitive information and removal of facial features, quality assurance on DICOM header and image information, and quantitative imaging biomarker extraction. In total, 1,616 examinations were selected based on the following MRI scanning protocols: dementia protocol (246), multiple sclerosis protocol (446) and open question protocol (924). We evaluated the effectiveness of the infrastructure in accessing and successfully extracting biomarkers from routinely acquired clinical imaging data. To examine the validity, we compared brain volumes between patient groups with positive and negative diagnosis, according to the patient reports. Overall, success rates of image data retrieval and automatic processing were 82.5 %, 82.3 % and 66.2 % for the three protocol groups respectively, indicating that a large percentage of routinely acquired clinical imaging data can be used for brain volumetry research, despite image heterogeneity. In line with the literature, brain volumes were found to be significantly smaller (p-value <0.001) in patients with a positive diagnosis of dementia (915 ml) compared to patients with a negative diagnosis (939 ml). This study demonstrates that quantitative image biomarkers such as intracranial and brain volume can be extracted from routinely acquired clinical imaging data. This enables secondary use of clinical images for research into quantitative biomarkers at a hitherto unprecedented scale.

## Introduction

In the last decades, the use of medical imaging in routine clinical practice has increased both in quantity and diversity. As advances in imaging hard- and software, and in imaging tracers, uncover new ways to visualize disease processes, medical imaging will continue to fulfill an important role in the diagnosis and treatment of patients in routine clinical care. The inclusion of imaging protocols in the diagnostic guidelines and criteria is a testimony of this importance. In the field of brain imaging for example, imaging has become critical for the diagnosis and/or operative planning for the treatment of acute traumas, tumors, and diseases such as epilepsy and multiple sclerosis, to name only a few. More recently, imaging has become supportive for the diagnosis of Alzheimer’s disease (Jack et al. 2011; Frisoni et al. 2010). Owing to these developments the amount of imaging data stored in Picture Archiving and Communication System (PACS) databases across hospitals continues to grow.

Traditionally, the interpretation of medical imaging data in clinical practice is performed qualitatively by trained radiologists. However, in clinical research and population studies, image information is increasingly condensed into a set of quantitative measures. This research is aimed at the development of ‘quantitative imaging biomarkers’, which objectively can determine the presence and stage of a disease or the response to a treatment. For example, the degree of carotid artery stenosis has been shown to be a quantitative imaging biomarker for stroke (NASCET 1991; ECST 1998). The Agatston score quantifies the amount of coronary calcification to predict the presence of obstructive coronary artery disease (Agatston et al. 1990; Kondos et al. 2003; Detrano et al. 2008). Also, a low hippocampal volume in magnetic resonance images has been used as quantitative imaging biomarker for dementia (den Heijer et al. 2006; Bobinski et al. 2000; Jack et al. 1992). Furthermore, computer tools are being developed to automate these measurements (Adame et al. 2004; Tang et al. 2012; Isgum et al. 2007; Shahzad et al. 2013; Fischl et al. 2002; van der Lijn et al. 2008).

Following both the trends of the increasing quantity of imaging data in routine clinical practice and the rising importance of quantitative imaging biomarkers in research, we see the natural opportunity for the secondary use of routinely acquired clinical imaging data to support medical imaging research on hitherto unprecedented scales. The principle of using legacy medical data for research has e.g. been successfully applied using electronic patient records from hospital databases (www.i2b2.org) or general practitioners databases (www.ipci.nl).

In recent years, the interest in using legacy imaging data for systematic medical knowledge discovery has increased. For example, information technology systems for unlocking clinical imaging data for research have been proposed based on the Biomedical Informatics Research Network (BIRN) (Chervenak et al. 2012; BIRN 2013), Extensible Neuroimaging Archive Toolkit (XNAT) (Doran et al. 2012; Marcus et al. 2007, 2011), Informatics for Integrating Biology & the Bedside (I2B2) (Mi2b2 2012; Murphy et al. 2010) or web-based infrastructures (Baltasar Sánchez and González-Sistal 2011; Bland et al. 2007). Furthermore, the feasibility of using these infrastructures for quantitative imaging research has been investigated. In the work by Hoogenboom et al. (2012, 2013), the feasibility of using biomarkers extracted from legacy imaging data has been investigated with brain morphology markers from structural magnetic resonance imaging (MRI) and white matter microstructure markers from diffusion tensor imaging (DTI). Also, Fennema-Notestine et al. (2007) demonstrated the feasibility of pooling legacy multi-center data into one analysis to investigate hippocampal changes in normal aging. In principle, a vast amount of information is contained in imaging data that are acquired in routine clinical care, but this information is not often used for research. The main advantages of using legacy data are the avoidance of additional acquisition costs and the potential to increase the scale of research. This could for example lead to the ability to detect subtle effects that may otherwise remain hidden and to capture rare cases. Moreover, it would allow for subdivisions within groups. However, there are serious challenges: logistical challenges related to secure data access, retrieval and anonymization, and data analysis challenges owing to heterogeneity in imaging data due to differences in scanner and acquisition protocols.

In view of this, the aim of this work is: 1) to design and implement a system which can extract imaging data from clinical repositories and process them such that they are available for research purposes; and 2) to show the feasibility to apply automated image processing to these data, for supporting quantitative imaging biomarker research on routinely acquired clinical imaging data. The system should satisfy the following requirements:

Anonymization: The first priority is to ensure the confidentiality of sensitive health care data. Secondary use of health care data requires consent, unless this is infeasible. If requesting consent is infeasible, data can be used for research if they are anonymized and proper safeguards are in place. In line with Directive 95/46/EC, the law for Protection of Personal data (Dutch: Wet Bescherming Persoonsgegevens), the Health Insurance Portability and Accountability Act and the World Medical Association Declaration of Helsinki (Gezondheidsraad 1993; KNAW 2003; HIPAA 2013; WMA 2002), proper de-identification mechanisms must be constructed and approved. Moreover, the infrastructure must be secure and guarded by a trusted and independent third party (privacy officer). This privacy officer provides the contact for patients with respect to their right to privacy and withdrawal.

Cope with clinical workflow: It is of utmost importance that the system does not interfere with the clinical workflow. The current infrastructure to access clinical images stored in hospital PACS databases is not suited for large scale image retrieval and processing. Available retrieval mechanisms could severely interfere with clinical workflow by overloading available resources. The system must also be able to cope with variation in scanning protocols, logistical issues and patient diversity. This requires a system which is robust to the input data and includes proper quality assurance algorithms.

Automation: The ease of use of the system for researchers will have a direct impact on the success of the system. The process of retrieving and processing routinely acquired data should be as automated as possible and should require little knowledge of the underlying components for successful execution. Furthermore, results must be easily reproducible and all details pertaining to how these results were obtained must be documented.

Flexibility: To fully exploit the diversity of imaging data acquired in routine clinical practice and to account for active research in image processing tools, the infrastructure must be open to improvements and alterations. This requires a flexible framework enabling changes in the various components to increase the effectiveness or to include other methods of quantitative imaging biomarker extraction.

## Infrastructure

**Fig. 1:**
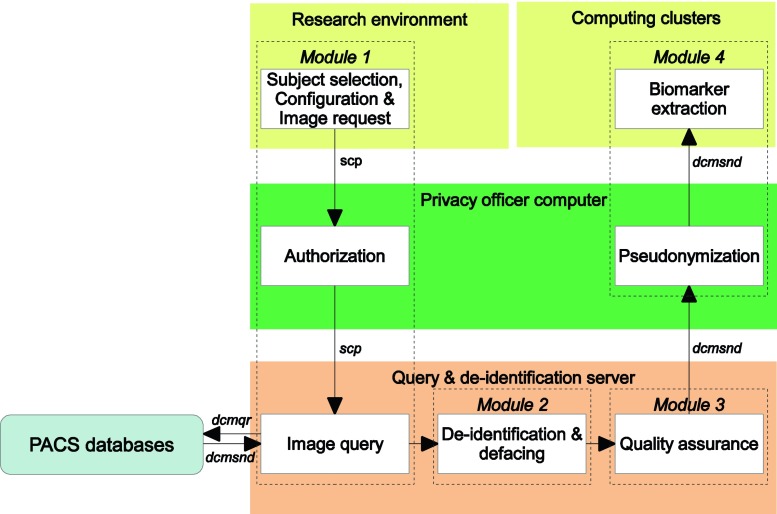
Schematic overview of the modular design of the infrastructure. Details of each module are described in the Infrastructure section

We designed and implemented an infrastructure taking into account the requirements above (Fig. 1). This infrastructure was approved by the Erasmus MC medical ethics committee and data protection authorities.

### Hardware

The infrastructure’s hardware consists of three main parts: a *query and de*-*identification server* connected to the PACS databases (2 × 6-core AMD Opteron 4180 2.6GHz 16GB), an authorization and pseudonymization computer under the supervision of the *privacy officer* (2 × 2-core Intel Xeon 5140 2.33GHz 14GB) and the research environment connected to *computing clusters* for fast quantitative imaging biomarker extraction (224 cores 2.1GHz AMD Opteron 6172 5GB per core). This design satisfies the requirements with respect to patient anonymization. The privacy officer computer is the only connection between patient data and the research environment. This allows the privacy officer to authorize and monitor all requests filed by researchers.

### Software

The system uses both publicly available programs and custom-made programs written in Java, Python or C++. Unix/Bash scripts were used to combine these programs into modules carrying out specific tasks. For example, for patient de-identification, software programs for the textual anonymization of DICOM files and for the defacing, using image registration, were combined into one module. The modular design enables a uniform way to supply input and output to the modules, and enables control over the parameter settings of the programs used within the module. The modules are connected by a single master Unix/Bash script to form a pipeline of data-processing stages. A configuration file can be supplied with parameter settings and paths which are propagated to the relevant programs and modules. This approach allows changes to individual modules without affecting the other modules while keeping the pipeline intact, or interchanging modules to make the infrastructure useful for other studies and applications. All developed software, including the modules used in this study and other modules which are used by other researchers in our group, will be provided under the lesser GNU Public License (LGPL) 3 through the Biomedical Imaging Group Rotterdam (BIGR) website (http://www.bigr.nl).

In the following sections, we will describe the modules in more detail. Alternative publicly available programs are listed for interested readers who want to replicate our approach.

### Module 1 Subject selection and PACS query

The interface to the system resides in the research environment. We have implemented a text-based search program to enable text mining on anonymized free text patient reports. This program is developed in java and access is restricted to authorized persons via a password. The program has an interface where a user can query the reports with regular expressions, upon which a list of study ID’s satisfying the search is returned. Possible search terms are for example MRI protocol codes, year of examination and other information which is routinely collected in clinical reporting.

Once a selection of study ID’s has been made, a request is sent via ‘scp’ to the privacy officer computer. Next to the list of study ID’s, this requests includes a configuration file, in which parameter settings can be provided, and the login information (user, password and ip-address). This action initiates a fully automated pipeline. The login information is checked with a simple matching program. For monitoring the requests, log files are automatically generated. The privacy officer has the possibility to halt all incoming requests or requests for individual patients via an exclusion list. The anonymous study-ID is mapped to the non-anonymous ID used in the hospital using a simple mapping program and mapping table. This mapping table has been previously generated when constructing the database of anonymized patient reports.

If the request is authorized, the request is passed on to the query and de-identification server. Querying the images from the PACS is achieved with a DICOM query (‘dcmqr’) on patient ID, a low-level operation which should be available in all PACS installations. DICOM series are sent within the infrastructure with ‘dcmsnd’. Both utilities are part of the DCM4CHE toolbox (http://www.dcm4che.org/), but can be substituted with appropriate alternatives which are also publicly available (e.g. tools which support C-find and C-store such as GDCM, http://gdcm.sourceforge.net/html/gdcmscu.html). Automization of the pipeline in this module and other modules is achieved with ‘storescp’, which is part of the OFFIS DCMTK toolkit (http://dicom.offis.de/dcmtk.php.en). This utility manages the arrival of DICOM images and initiates further steps.

### Module 2 De-identification and defacing

After the images are retrieved, the images are de-identified on a textual and image level. Imaging data in PACS are stored in DICOM format (Digital Imaging and Communications in Medicine). The header of each DICOM file contains textual information essential for image identification, which includes patient sensitive information such as names, locations, identifiers and dates. Additionally, scan vendors and technicians have the possibility to enter additional information in private entries. Therefore, all patient sensitive elements specified in the DICOM specification (Medical Imaging & Technology Alliance 2011) are removed, together with all the private elements, as it cannot be foreseen whether patient information is stored in private fields. Birth dates are reset to January 1st of the year of birth. We employed DICOM libraries implemented in Python (http://code.google.com/p/pydicom/) to remove and change DICOM entries (an alternative is the ‘dcmodify’ tool in the OFFIS DCMTK).

In case of brain images, de-identification is further accomplished via a defacing algorithm which automatically removes facial features from images of the head. Details of this step are explained in the ‘Experiments’ section. Defacing was carried out by registering a mask to the image using the Elastix registration program (Klein et al. 2010). The registered mask does not contain the facial area. Thus by multiplying the image with this mask, we remove the facial features. The software is publicly available, and we provide the parameters we used on the Elastix website. Viable alternatives include FLIRT 2013, which is a publicly available tool from FSL (http://fsl.fmrib.ox.ac.uk/fsl/fslwiki/FLIRT).

### Module 3 Quality assurance

After de-identification and defacing, images are automatically checked by quality assurance algorithms to ensure that they are of sufficient quality to extract the quantitative imaging biomarkers of interest. Both textual information, contained in the DICOM headers, and image features are utilized in this step.

Using the DICOM header entries, we first determine the type of MRI scan sequence from the image acquisition parameters. A Python program has been written to classify MR images based on e.g. the echo time, inversion time, repetition time and flip angle. Reported values in literature were used to determine the classification (Jackson et al. 1997; Bernstein et al. 2004; Westbrook 1999). Using this Python program, we can impose requirements on the image such as the aforementioned scan sequence classification, but also other DICOM entries can be used such as the resolution, slice thickness and contrast agent. This program is available on the BIGR website.

We also check whether the image volume contains the full anatomy of interest. For this task, also image registration is used; details of this step are provided in the Experiments section for brain imaging, but the methodology can readily be applied to other anatomical regions.

### Module 4 Quantitative imaging biomarker extraction

Image datasets that successfully passed quality assurance are sent to the privacy officer computer for pseudonymization, i.e. the non-anonymous ID used in the hospital is mapped back to the anonymous study (pseudo-)ID used in the research setting. This mapping is only available at the privacy officer and can be used for linkage when performing studies combining multiple health care data sources. The same mapping program is used as in module 1.

The fully anonymized images are sent to the research environment for quantitative imaging biomarker processing. For MRI brain images, prior to biomarker extraction we preprocessed the images with non-uniformity correction (N4itk: Tustison et al. 2010) and conversion to the NifTI image format (http://www.mccauslandcenter.sc.edu/mricro/mricron/dcm2nii.html). We used a brain tissue segmentation method which was developed in our own lab (Vrooman et al. 2007). Alternative packages are publicly available, e.g. Freesurfer (http://freesurfer.net/fswiki/SubcorticalSegmentation), Fast (http://fsl.fmrib.ox.ac.uk/fsl/fslwiki/FAST), or SPM (http://www.fil.ion.ucl.ac.uk/spm/), which are widely used in the research community. The latter two methods have been compared to the segmentation method used in this infrastructure and it has shown that the methods achieve similar performance (de Boer et al. 2010).

## Experiments

As a proof of principle study we performed intracranial volume and brain tissue volume extraction from routinely acquired clinical imaging data. This allows us to study patterns of atrophy in different age and patient groups. Such studies have so far been primarily carried out in well-defined prospective population studies or clinical cohort studies. For example, population studies have provided information on grey matter and white matter atrophy with age (Ikram et al. 2007; Sigurdsson et al. 2012). In frontotemporal dementia (Lund and Manchester Groups 1994; Neary et al. 1998; Rascovsky et al. 2011) and Alzheimer’s disease (Dubois et al. 2007, 2010; McKhann et al. 2011), atrophy of respectively frontal lobes and medial temporal lobe regions have been reported and included in diagnostic criteria. Also multiple sclerosis (MS) has been associated with brain atrophy (Ge 2006; Fischer et al. 2008; Bakshi et al. 2005). The importance of imaging in the diagnostic process of these diseases has resulted in a large amount of routine clinical data available for this pilot study (Jack et al. 2011; Frisoni et al. 2010; McDonald et al. 2001; Polman et al. 2011).

### Research Questions

In the proof of principle study of the infrastructure, we investigate the operational performance and the potential of the secondary use of clinical imaging data. The experiments were designed to address two research questions:

1. What are the success rates of the infrastructure in accessing routinely acquired imaging data and subsequent quantitative imaging biomarker extraction?
2. Can we provide a proof of principle that the system is a useful research tool in associating imaging biomarkers with other parameters such as gender, age and disease?

### Patient Selection and Configuration

Image selection was based on scanning protocol codes and date of acquisition. Imaging data were selected from PACS databases of the Erasmus MC, University Medical Center Rotterdam. We restricted our analysis to the period from January 2007 to January 2012, during which a total of 21.998 brain MRI scans were acquired. We employed text mining on the radiology reports to extract imaging studies performed under the dementia (246 subjects), MS (446) and open question (924) protocols. The dementia and MS protocols have been included to address the second research question. The open question protocol is used if patients exhibit a-specific neurological symptoms. This protocol group contains patients with a large diversity both in disease and scanning practices, and will help to evaluate the robustness of the infrastructure for a wide range of imaging data.

The scanning protocol codes used for patient selection are part of the clinical workflow and indicate the purpose of scanning. As the protocol code is assigned to an examination prior to the diagnosis, it may not be used to assign a patient to a disease group. Therefore, we reviewed the patient records to control for the diagnosis of dementia and MS. The positive diagnosis of dementia was assigned if the report mentioned the diagnosis of dementia as determined by a specialist or if findings which are indicative of dementia (lobar, cortical or hippocampal atrophy, enlarged ventricles and white matter abnormalities) as determined by a trained radiologist. For the positive diagnosis of MS we verified whether the report contained a confirmed diagnosis by a specialist, or a pathology satisfying the McDonald criteria; dissemination of space and time of white matter lesions periventricular, juxtacortical, infratentorial and/or in the spinal cord (Polman et al. 2011). The negative diagnosis was assigned if the diagnosis was ruled out by a specialist or if no findings satisfying the criteria were reported. This procedure resulted in 115 subjects with a positive diagnosis of dementia and 131 subjects with a negative diagnosis. For the MS subjects, 241 subjects were positively diagnosed versus 173 negatively diagnosed. The negatively diagnosed patients are scanned using the same set of scanners and are hence used in our study as a control group for the positively diagnosed patients.

The image processing pipeline requires MR T1 weighted (T1w) images, or if not available, PDw and T2w scans of the head without contrast agent. Contrast agent such as gadolinium often appears hyperintense in MR images, which could lead to misclassifications in tissue volumes.

### De-Identification

**Fig. 2:**
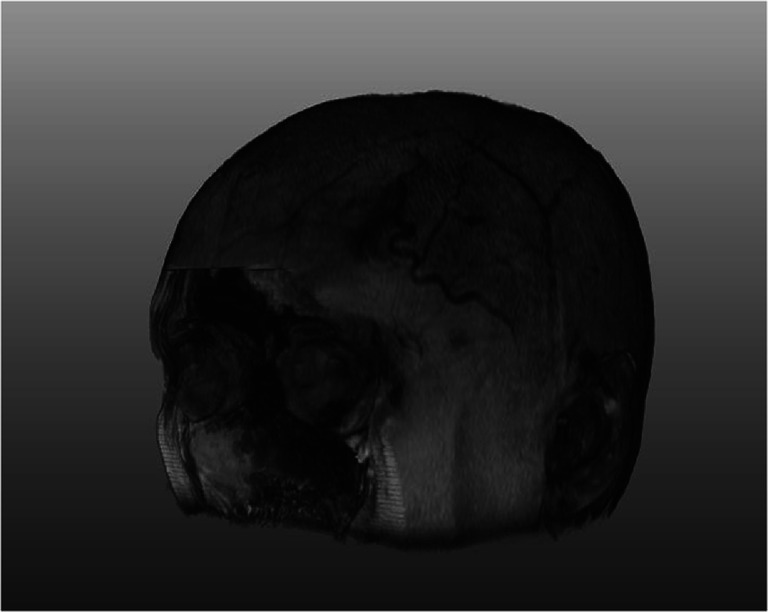
An example output of the defacing algorithm, which removes facial features from images using image registration

All patient sensitive information was removed from the DICOM headers, e.g. names, locations, identifiers and comments. Furthermore, as the images used in this study contained the face, we used atlas registration (Klein et al. 2010) to mask the facial features. The method is based on atlas registration with the MNI152 atlas (Fonov et al. 2009; 2011). Using affine image registration, the atlas image is deformed in such a way that it is similar to the target image, establishing spatial correspondence between points in the atlas and target image. In the atlas image, an operator manually defined a region of interest excluding the facial features (atlas label image). By applying the same deformation to the atlas label image we masked the subject image. An example is provided in Fig. 2.

### Quality Assurance

Images were first classified into different MRI sequences (e.g. T1w, T2w, PDw, diffusion weighted and functional MR images) based on image acquisition parameters in the DICOM header to remedy the variability in naming. Images retrieved from PACS were filtered on the MRI sequence specifications in the configuration file and the highest resolution image of each scan sequence was selected for subsequent processing.

As part of the automatic quality check of the incoming data, it was determined whether the image contains the full extent of the brain. In this step we again used the MNI152 atlas (Fonov et al. 2009; 2011) which was affinely registered to the subject image. The MNI152 brain mask was transformed to subject space, so as to label the voxels belonging to the brain. If more than 20 % of the voxels on one of the outer image planes are labeled as brain, it is likely that the full brain ROI is not contained in the image, and the image is excluded.

### Quantitative Imaging Biomarker Extraction

Images were preprocessed by MRI bias field correction to correct for slowly varying signal intensity differences (Tustison et al. 2010). For brain tissue segmentation, a k-Nearest Neighbor (kNN) method is used, which is trained on the subject to be segmented (Cover and Hart 1967). For a detailed overview of the method we refer to Vrooman et al. (2007). Briefly, the method relies on six atlas images (in which cerebral spinal fluid, grey matter and white matter have been outlined by a radiologist), which are non-rigidly registered to the subject image to be segmented by maximizing mutual information (Pluim et al. 2003). The deformations resulting from this registration step are then applied to the corresponding atlas label image. Averaging the transformed atlas label images produces a probability map of brain tissues for the target image, which can be interpreted as the probability of voxels belonging to a certain tissue class. Using the probability map, likely samples of the different classes are extracted from the target image and used to build the intensity based kNN-classifier.

The advantage of this approach of atlas-based training is that possible differences in contrasts between the atlas and target image are dealt with in the registration step and do not affect the segmentation. In addition, since we utilize mutual information as similarity measure in the registration, it is possible to register the atlases to images obtained with a different scanning protocol. For example we are able to use T1-weighted atlas images to segment brains on T2- or PD-weighted images.

**Fig. 3:**
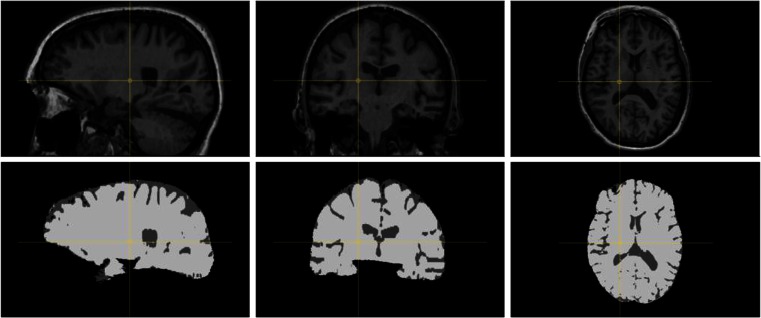
Fig. 3 T1-weighted image (*first row*) and result after segmentation in brain tissue and CSF (*second row*). The brain is depicted in sagittal, coronal and axial orientation

The images are segmented into cerebral spinal fluid (CSF), grey matter (GM), white matter (WM) and background. Whole brain volume is formed by the sum of GM and WM. Intracranial volume (ICV) is determined by the sum of CSF, GM and WM. Summing all voxels (volume elements, 3D pixels) of a certain tissue yields tissue volumes in milliliters. Skull masking was performed by multi-atlas registration. Similar to the quality assurance step, multiple (6) non-rigidly registered atlas brain label images are combined into a single brain mask by majority voting (with a threshold of 0.8). The cerebra (excluding cerebellum) in the 6 atlas images were manually annotated by an expert neuroradiologist. Figure 3 shows a typical segmentation result.

### Data Analysis

#### Performance

To evaluate the performance of the infrastructure in accessing routinely acquired imaging data and subsequent quantitative imaging biomarker extraction, we report the success rates at the following steps in the processing pipeline: i) image query; ii) quality assurance; and iii) quantitative imaging biomarker extraction. The final segmentations are visually inspected in the three orthogonal mid-planes to verify the segmentation results. This last step (iv) is aimed to investigate whether visual inspection is mandatory in future studies, or whether fully automatic biomarker research on routinely acquired clinical imaging data is feasible.

#### Proof of Principle

To provide a proof of principle that the infrastructure can reproduce known results from literature, we first selected all patients which at time of scanning were 45 years or older. This selection was applied to make the age range of the different groups more comparable. We employed an automatic outlier exclusion criterion to exclude biomarkers with largely deviating volumes. We excluded all subjects with ICV corrected brain volumes outside [Q_1_-1.5*IQR; Q_3_ + 1.5*IQR], where Q_n_ and IQR denotes the nth quartile and the interquartile range respectively. To provide more insight in the heterogeneity of the imaging data in different groups, we present the image acquisition parameters in Table 2.

**Table 1:** Characteristics of the study population, stratified by gender and group. Note that these numbers are obtained after the age selection of 45 years and older and the automatic outlier exclusion criterion

		Total	Men	Women
Dementia (positive diagnosis)	Number	83	48	35
	Age (years)	67.5 ± 8.4	68.5 ± 8.4	66.1 ± 8.5
Dementia (negative diagnosis)	Number	79	39	40
	Age (years)	60.5 ± 8.3	60.2 ± 8.1	60.7 ± 8.6
Multiple sclerosis (positive diagnosis)	Number	76	27	49
	Age (years)	54.4 ± 7.4	54.1 ± 7.8	54.5 ± 7.3
Multiple sclerosis (negative diagnosis)	Number	70	35	35
	Age (years)	58.3 ± 10.4	59.9 ± 10.6	56.7 ± 10.0
Open question	Number	344	190	154
	Age (years)	60.7 ± 9.8	60.5 ± 9.2	61.1 ± 10.5

Age distributions are described by mean and standard deviations in years

**Fig. 4:**
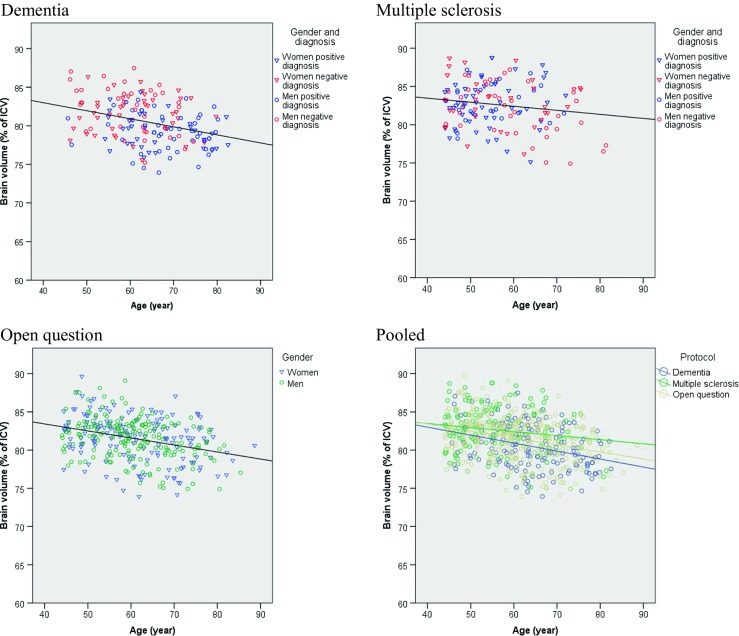
Scatter plots of brain volumes versus age, segregated by gender (Men: *open circles*. Women: *open triangles*). For the dementia and multiple sclerosis protocol groups, blue denotes a positive diagnosis and red a negative diagnosis. Regression lines for linear fit are shown for men and women combined. Results are presented for the three protocol groups: dementia, multiple sclerosis and open question respectively (after age ≥45 year and IQR selection). The last plot combines all protocol groups. Volumes are expressed as percentage of intracranial volume

We plotted quantitative imaging biomarkers (brain volumes as percentages of ICV) as function of age for different subgroups and gender (Fig. 4). For the statistical analyses, we used linear regression models to investigate demographic (gender/age), disease group and scanner effects:

1 $$ ICV={\beta}_0+{\beta}_1 gender+{\beta}_2 age+{\displaystyle \sum_{i= subgroups}}{\beta}_{3 i} grou{p}_i+{\beta}_4 seq+{\displaystyle \sum_{j= scanner}}{\beta}_{5 j} typ{e}_j+{\beta}_6 fs+\varepsilon $$

In this equation, the nominal variable ‘group’ denotes the different disease subgroups (positive and negative diagnosis of dementia and MS and the open question group). Combining images from different sources can increase power due to a larger sample size, but the likely added variability must properly be taken into account. Therefore, we added the scanning sequence (seq), the scanner manufacturer and model type (type) and the magnetic field strength (fs) into the model to investigate this effect. The possible values for the scanner hardware and scan sequence parameters are listed in Table 2. We employed the conventional α = 0.05 level for significance. For brain volumes we added ICV as additional effect to control for differences in head sizes:

2 $$ Brain\  volume={\beta}_0+{\beta}_1 gender+{\beta}_2 age+{\displaystyle \sum_{i= subgroups}}{\beta}_{3 i} grou{p}_i+{\beta}_4 seq+{\displaystyle \sum_{j= scanner}}{\beta}_{5 j} typ{e}_j+{\beta}_6 fs+{\beta}_7 ICV+\varepsilon $$

All statistical analyses were performed using SPSS version 20.

## Results

### Performance

**Fig. 5:**
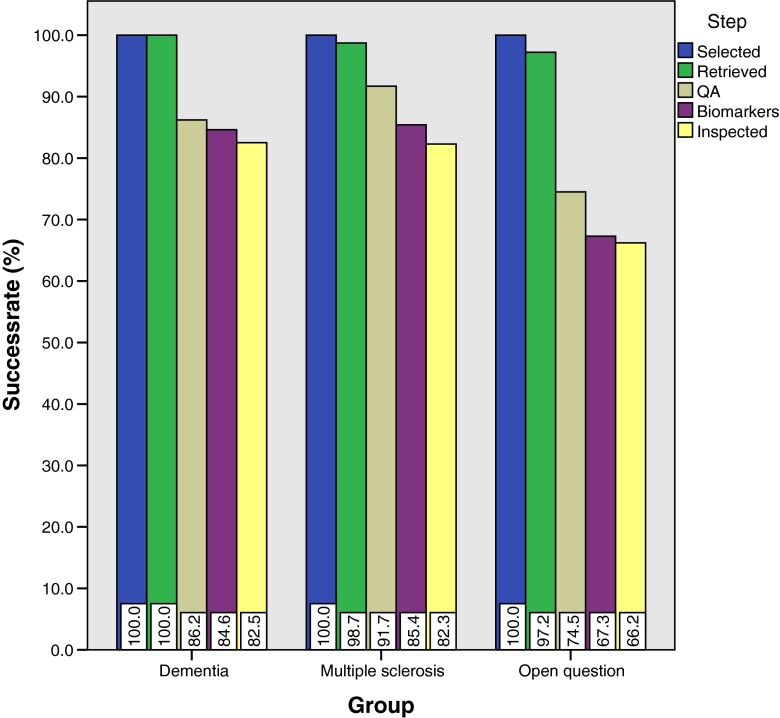
Success rates (%) at different steps in the processing pipeline shown for the three study populations. Starting number of subjects are *n* = 246, 446 and 924 for the dementia, multiple sclerosis and open question groups

The success rates at the different steps in the automated pipeline are shown in Fig. 5 as percentages of the number of patients at selection.

Step i Across all protocol groups we observed high success rates (>97.2 %) for data retrieval from the PACS database.
Step ii For the dementia and MS protocols, 86.2 % and 91.7 % passed the quality assurance test, respectively. The open question group showed a lower success rate (74.5 %). Inspection showed that this can be attributed to the more frequent deviation from protocol, such as the inclusion of contrast agent (150 cases) or limited field of view (57), situations which lead to exclusion in our study design. In the dementia protocol group, 19 subjects were excluded due to incomplete field of view and 25 for inclusion of contrast agent. In the MS group, the numbers for exclusions were 12 and 19 respectively.
Step iii The extraction of quantitative imaging biomarkers was successful in most instances, independent of the protocol code. The number of images that passed the whole chain was reduced by 7.2 % or less. Errors were often due to severe pathology in patients with large tumors, head traumas, severe atrophy or large resections.
Step iv After visual inspection final success rates were 82.5 % 82.3 % and 66.2 % for the dementia, multiple sclerosis and open question protocol groups respectively. The small decrease compared to the previous step shows that only a small percentage of images passing biomarker extraction yielded faulty segmentations.

The processing time involved from image query to completion of biomarker extraction in all 1616 subjects combined was 40 h.

### Proof of Principle

To show the potential of automated quantitative imaging biomarker extraction from routinely acquired imaging data, we investigate whether we can reproduce findings that have been reported in literature. After excluding the subjects not satisfying the age range of 45 years and older and the automatic outlier exclusion criterion based on the IQR, the characteristics of this study population are listed in Table 1.

The image acquisition parameters of the MR images can be seen in Table 2. The second column shows the distribution of scanner types for each group. The subsequent columns show the distribution of image acquisition parameters which influence image resolution and contrast, taken for all scanner types combined and stratified per group.

**Table 2:** Image acquisition parameters of the MR images used in the statistical analyses

	Scanner type	Image resolution					Image contrast					
		Field strength	Slice thickness (mm)	Matrix	Pixel size (mm^2^)	Number of slices	Scanning sequence	Repetition time (s)	Echo time (ms)	Inversion time (s)	Flip angle (degrees)	Pixel bandwidth (Hz/pixel)
Dementia (positive diagnosis) *n* = 83	GE Discovery, 67 (81 %) GE Signa, 6 (7 %) Siemens Sonata, 10 (12 %)	1.5 T 20 (24 %) 3.0 T 63 (76 %)	1.0 (1.0-1.5)	240 × 240, 60 (72 %) 320 × 191, 10 (12 %) 416 × 256, 6 (7 %) 256 × 256, 4 (5 %) …	0.8 (0.3–0.8)	176 (158–176)	GR 82 (99 %)	7.9 (7.8–7.9)	3.1 (3.1–3.1)	450 (450–450)	12 (12–12)	244 (122–244)
							SE 1 (1 %)	437	10.1	0	90	122
Dementia (negative diagnosis) *n* = 79	GE Discovery, 29 (37 %) GE Signa, 21 (27 %) Siemens Sonata, 28 (35 %) Philips NT Intera, 1 (1 %)	1.5 T 58 (73 %) 3.0 T 21 (27 %)	1.6 (1.0–5.0)	240 × 240, 18 (23 %) 384 × 224, 13 (17 %) 256 × 132, 9 (11 %) 256 × 256, 7 (9 %) 320 × 191, 7 (9 %) …	0.3 (0.3–0.8)	150 (26–176)	GR 49 (54 %)	7.9 (7.9–15.0)	3.1 (3.0–4.8)	450 (450–450)	12 (12–23)	170 (122–244)
							SE 62 (38 %)	565 (560–577)	12.0 (11.2–14.0)	0 (0–0)	90 (90–90)	94 (90–98)
Multiple sclerosis (positive diagnosis) *n* = 76	GE Discovery, 9 (12 %) GE Signa, 22 (29 %) Siemens Sonata, 14 (18 %) Philips NT Intera, 31 (41 %)	1.5 T 68 (90 %) 3.0 T 8 (10 %)	5.0 (5.0–5.0)	256 × 192, 23 (30 %) 256 × 132, 6 (8 %) 416 × 384, 6 (8 %) 256 × 144, 3 (4 %) …	0.8 (0.8–0.8)	24 (23–26)	GR 4 (5 %)	14.0 (9.6–15.8)	4.8 (3.6–8.7)	450 (450–450)	23 (15–28)	170 (122–170)
							SE 72 (95 %)	2500 (2,420–2,652)	15.0 (8.0–20.0)	0 (0–0)	90 (90–90)	122 (122–163)
Multiple sclerosis (negative diagnosis) *n* = 70	GE Discovery, 4 (6 %) GE Signa, 41 (59 %) Siemens Sonata, 12 (17 %) Philips NT Intera, 13 (19 %)	1.5 T 65 (93 %) 3.0 T 5 (7 %)	3.5 (2.0–5.0)	256 × 224, 17 (24 %) 256 × 192, 12 (17 %) 256 × 132, 8 (11 %) 416 × 256, 8 (11 %) …	0.8 (0.7–0.8)	35 (23–74)	GR 28 (40 %)	17.1 (13.4–17.1)	7.6 (2.5–7.6)	0 (0–400)	30 (20–30)	75 (70–75)
							SE 42 (60 %)	2,480 (2,440–2,652)	15.0 (8.0–20.0)	0 (0–0)	90 (90–90)	122 (122–195)
Open question *n* = 344	GE Discovery, 61 (18 %) GE Signa, 67 (20 %) Siemens Sonata, 58 (17 %) Philips NT Intera, 158 (46 %)	1.5 T 295 (86 %) 3.0 T 49 (14 %)	2.0 (1.5–2.0)	256 × 224, 39 (11 %) 256 × 157, 34 (10 %) 256 × 256, 25 (7 %) 240 × 240, 20 (6 %) 416 × 256, 18 (5 %) …	0.8 (0.24–0.8)	70 (64–144)	GR 302 (86 %)	16.3 (13.4–16.5)	7.6 (3.7–10.0)	400 (0–450)	30 (20–30)	170 (75–217)
							SE 42 (14 %)	513 (500–564)	12.7 (10.0–14.0)	0 (0–0)	90 (90–90)	98 (90–122)

Values represent number (%) or median(lower quartile-upper quartile). Abbreviations: *GR* gradient echo, *SE* spin echo

### ICV

#### Effect of Gender and age on ICV

**Fig. 6:**
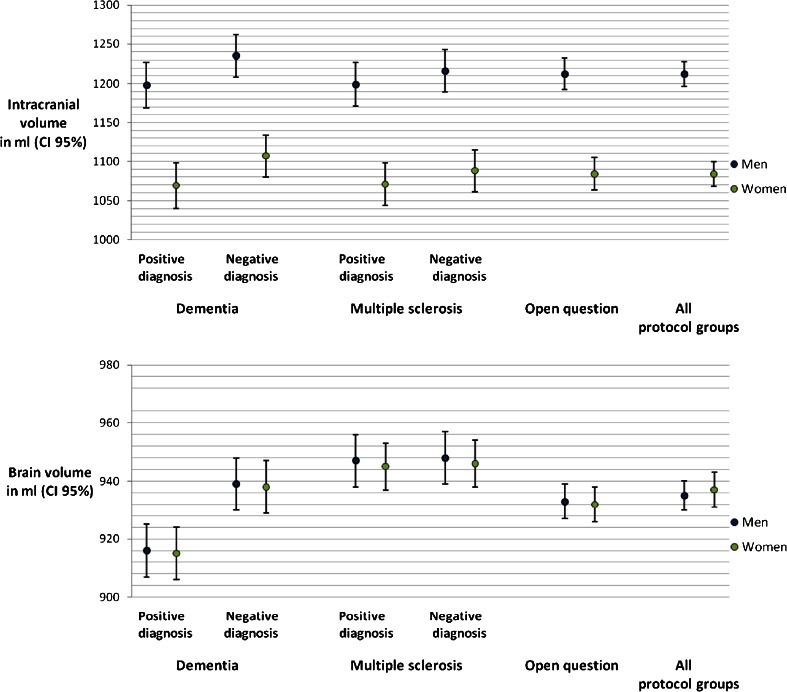
Intracranial and brain volumes stratified by gender and group. Multiple linear regression: all analyses are corrected for age and scanner effects, Brain volumes are corrected for ICV, the covariates are evaluated at age = 60.5, ICV = 1,150 ml, and the Philips NT Intera is taken as reference. Analyses on the total pooled group are corrected for the individual groups

The linear regression model for ICV (Equation 1) yielded an adjusted R^2^ = 31.0 % and F(11,640) = 27.6, *p* < 0.001. Gender accounted for 28.7 % of this variance. ICV values stratified by gender are shown in Table 3 and Fig. 6. The model showed significant ICV differences in gender in the total population (t(640) = 16.4, *p* < 0.001) and in the individual groups (positive dementia t(76) = 4.95; negative dementia t(71) = 5.28; positive MS t(62) = 5.69; negative MS t(62) = 5.05; open question t(336) = 12.7; all *p* < 0.001). Men had larger ICV than women.

**Table 3:** Intracranial and brain volume (corrected for ICV) stratified per group and gender

	ICV in ml (CI 95 %)			Brain volume in ml (CI 95 %)		
	Men	Women	Total	Men	Women	Total
Dementia (positive diagnosis)	1,198 (1,169;1,227)	1,069 (1,040;1,099)	1,134 (1,105;1,162)	916 (907;926)	915 (906;924)	915 (907;924)
Dementia (negative diagnosis)	1,235 (1,208;1,262)	1,107 (1,080;1,134)	1,171 (1,145;1,197)	930 (931;948)	938 (929;947)	939 (930;947)
Multiple sclerosis (positive diagnosis)	1,199 (1,171;1,228)	1,071 (1,044;1,098)	1,135 (1,108;1,162)	947 (938;956)	945 (937;955)	947 (938;955)
Multiple sclerosis (negative diagnosis)	1,216 (1,189;1,244)	1,088 (1,061;1,116)	1,152 (1,126;1,179)	948 (939;957)	946 (938;955)	947 (939;956)
Open question	1,212 (1,192;1,232)	1,084 (1,063;1,104)	1,148 (1,129;1,166)	933 (927;940)	932 (926;939)	933 (927;939)
Total pooled	1,212 (1,196;1,228)	1,084 (1,068;1,100)		935 (930;941)	937 (931;942)	

Multiple linear regression: all analyses are corrected for age and scanner effects, Brain volumes are corrected for ICV, the covariates are evaluated at age = 60.5, ICV = 1,150 ml, the Philips NT Intera scanner is taken as reference. Analyses on the total pooled group are corrected for the groups

**Fig. 7:**
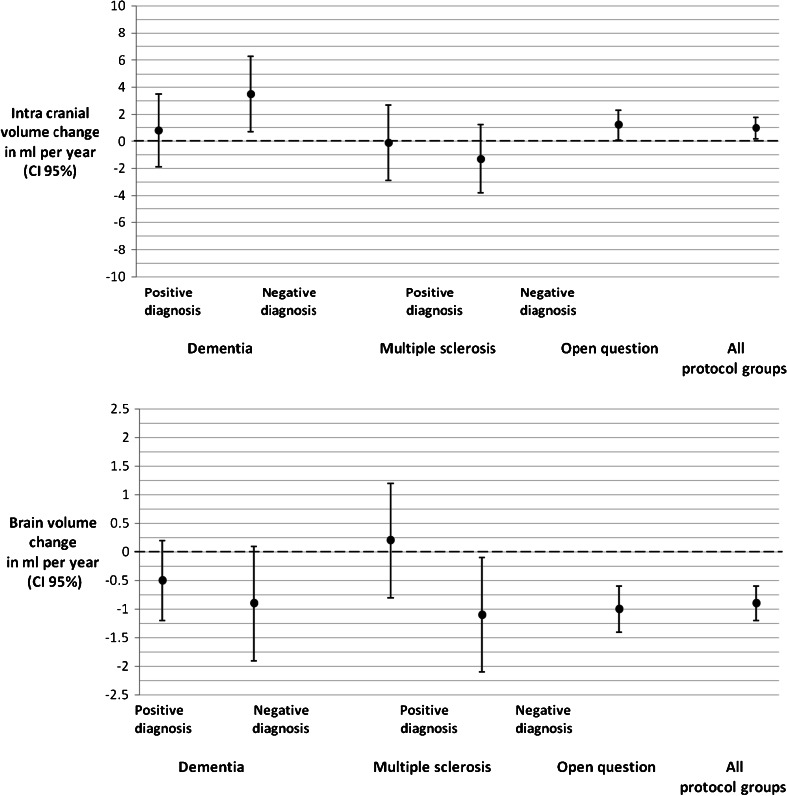
Intracranial and brain volumes versus age. Multiple linear regression: all analyses are corrected for scanner effects, Brain volumes are corrected for ICV, Analyses on the total pooled group are corrected for the individual groups

As expected, age had no large effect and only explained 0.4 % of the variance. A small but statistically significant increase in ICV was found (Table 4 and Fig. 7), which in the total population was 1 ml/year (t(640) = 2.4; *p* = 0.019). In the individual groups, the negatively diagnosed dementia group and open question group showed a significant increase in ICV (t(71) = 2.54, *p* = 0.01; t(336) = 2.19, *p* = 0.03 respectively). However, this was a very small effect compared to the mean ICV and likely caused by the heterogeneity of the clinical data.

**Table 4:** The relationship between intracranial volume (ICV) and brain volume (corrected for ICV) with age

	ICV	Brain volume
	Age predictor	Age predictor
	β in ml per year (CI 95 %)	β in ml per year (CI 95 %)
Dementia (positive diagnosis)	0.8 (−1.9;3.5)	−0.5 (−1.2;0.2)
Dementia (negative diagnosis)	3.5 (0.7;6.2)	−0.9 (−1.9;0.2)
Multiple sclerosis (positive diagnosis)	−0.1 (−2.9;2.7)	0.2 (−0.8;1.2)
Multiple sclerosis (negative diagnosis)	−1.3 (−3.8;1.2)	−1.1 (−2.1;−0.2)
Open question	1.2 (0.1;2.4)	−1.0 (−1.4;−0.7)
Total pooled	1.0 (0.2;1.8)	−0.9 (−1.2;−0.6)

Multiple linear regression: all analyses are corrected for gender and scanner effects. Brain volumes are corrected for ICV. Analyses on the total pooled group are corrected for the groups

#### Effect of Groups on ICV

Group label had no large effect and only explained 0.3 % of the variance of the data. In Table 3, the difference in ICV between groups is shown. The linear regression model did not show any significant effects (all *t* < 1.75, *p* > 0.08).

#### Effect of Scanner Parameters on ICV

Scanner parameters accounted for 0.7 % of variance in the data. The model did not show any significant effects on ICV due to the scanning sequence (t(640) = 0.97, *p* = 0.33), scanner type (|t(640)| < 1.24, *p* > 0.22) or field strength (|t(640)| = 1.79, *p* = 0.07).

### Brain Volume

#### Effect of Gender and age on Brain Volume

The linear regression model for brain volumes (Equation 2) yielded an adjusted R^2^ = 90.7 % and F(12,639) = 532, *p* < 0.001. In addition to the previous model, we added ICV as effect which had a highly significant contribution (t(639) = 65.4, *p* < 0.001) and explained 61.9 % of the variance. Gender did not explain any variance. Brain volumes are shown in Table 3 and Fig. 6 for the different genders. Between men and women, we did not find any significant effect for gender in the total population (|t(639)| = 0.44, *p* = 0.66) or in the individual groups (|t(639)| < 0.98, *p* > 0.33). Men and women did not differ in brain volume when correcting for ICV.

Age explained 0.6 % of the variance. We found a significant age effect on brain volume (t(639) = −6.72, *p* < 0.001) signifying a decrease in brain volume with age in the total population. This effect was significant in the open question and negative MS groups (t(61) = −2.38, *p* = 0.02; t(335) = −5.93 *p* < 0.001 respectively).

#### Effect of Groups on Brain Volumes

Group label explained 0.4 % of the variance of the data. Brain volumes corrected for ICV differed significantly among the five groups (F(12,639) = 7.77, *p* < 0.001). Post hoc analyses with Bonferroni correction revealed that the difference between the positively and negatively diagnosed dementia groups was highly significant (t(639) = 4.26, *p* < 0.001) and that positively diagnosed dementia patients had smaller brain volumes. Between the positive and negative MS groups the brain volumes did not differ significantly (t(639) = 0.087, *p* = 0.93).

#### Effect of Scanner Parameters on Brain Volume

The variation in scanner parameters accounted for 0.1 % of the data variance. The model did not show any significant effects on brain volume due to the scanning sequence (t(639) = 1.73, *p* = 0.08), scanner type (|t(639)| < 1.3, *p* > 0.19) or field strength (|t(639)| = 1.08, *p* = 0.28).

## Discussion

We have presented an infrastructure to support the secondary use of routinely acquired clinical imaging data for research. This system is capable of extracting routinely acquired imaging data from PACS databases, satisfying requirements with respect to legal issues (anonymization) and logistical issues (automation and quality assurance), and we showed in a proof of principle study that the system is a useful research tool for associating imaging biomarkers with other parameters such as gender, age and disease. Although the secondary use of electronic patient records for research is well established, we have not encountered a feasibility study and quantitative evaluation of using legacy clinical imaging data on the scale of a thousand subjects before in literature.

### Relationship to Literature

Hoogenboom et al. (2012, 2013) and Fennema-Notestine et al. (2007) have previously reported on quantitative evaluations of the use of multi-scanner legacy imaging data for quantitative biomarker research. In their work, they investigated extraction of more sophisticated quantitative imaging biomarkers (subcortical structures and DTI measures) on images that satisfied certain quality criteria, such as field strength, maximum slice thickness, image acquisition orientation, and minimum brain tissue contrast. Applying these quality criteria resulted in a smaller data set. For example, in the work by Hoogenboom et al. (2012) 20 out of 320 images were retained after quality criteria.

We foresee that when using legacy clinical imaging data there will be a trade-off between the complexity of the imaging biomarker and the number of data that satisfy the quality criteria to be included in the analysis. Fairly gross quantitative biomarkers such as brain volume can be extracted from a large percentage of the legacy data, as is shown in this work, whereas more sophisticated quantitative imaging biomarkers can probably only be extracted from a smaller percentage of datasets. We believe that both methods are very useful for opening legacy imaging data for research, and that they complement each other.

As we have encountered in this study, legacy clinical imaging data is often acquired in a multi-scanner setting with a variety of acquisition settings and protocols. Recently, there are significant efforts into standardizing imaging protocols across scanners to reduce heterogeneity, which would greatly facilitate the sharing of imaging data across institutes (Jack et al. 2008; Glover et al. 2012). However, owing to differences between scanner vendors, technology development, and the fact that the majority of imaging data in clinical routine is currently not part of standardized multicenter trials, the development of robust image processing tools also remains an important key to address this problem.

Therefore, we think that the problem of heterogeneity of imaging data is to be dealt with from two sides, both standardization of scanning protocols, and the development of robust standardized image processing tools that can deal with data heterogeneity. Many image analysis tools have been designed and optimized on a limited data set. Apart from a handful of publicly available image processing toolboxes, not many tools are being applied outside their own research environment. We think that there is a need for imaging tools that have been tested on a variety of imaging data, to ensure that they are robust for a wide range of images and to enable the use in different institutes. Therefore we also welcome the concepts of challenges on multicenter and multivendor data, to objectively compare the performance of algorithms in different settings (Grand challenges in medical image analysis 2013: http://grand-challenge.org/All_Challenges/).

### Discussion of the Results

The evaluation of the proposed infrastructure’s performance has yielded promising results; the system is able to unlock and process high percentages of legacy clinical imaging data for research. Especially considering the dementia and multiple sclerosis group, over 82.3 % of available data was useable in this study. Although the success rate was lower for the open question group, this was mainly due to exclusion in the quality assurance step. We identified two main causes, namely contrast agent in the image and an incomplete field of view. As these cases would have led to untrustworthy biomarkers, the successful identification by the quality assurance algorithms indicates good performance by the system, despite a lower final success rate. The small drop in success rate from biomarker extraction to visual inspection indicates that a large percentage of obtained biomarkers are usable for research and that fully automatic biomarker research on routinely acquired clinical imaging data is feasible.

The proof of principle study showed that we could replicate a number of findings which have previously been reported in literature. We found ICV to be larger for men, an association which has also been reported in large studies on the elderly population, e.g. Ikram et al. 2008 and Sigurdsson et al. 2012.

In our investigation of the effect of age, we found ICV to increase significantly in our open question group and negatively diagnosed dementia subgroup. Although this is an unexpected finding, the effect is not large (smaller than 0.1 % of the mean ICV) and might still be caused by data heterogeneity.

We did not find any significant effect of group label or scanner parameter in our ICV data. This is encouraging as we do not expect a correlation between head size and disease. Furthermore, our findings thus do not show that heterogeneity in scanner types, scan sequences or magnetic field strength affect automatic extraction of ICV.

Differences in ICV corrected brain volume due to gender were not found in our study. A number of studies have found ICV corrected brain volumes larger in women (Ikram et al. 2008; Sigurdsson et al. 2012). In our data we see the same trend. This was, however, not statistically significantly.

We also found statistically significant associations between brain volume and age in the open question group and negative MS group, which could be attributed to normal aging. The absence of such an age effect on brain volume in the positively diagnosed groups may be caused by confounding by disease severity in the patient groups. Patients undergo MRI examination at a certain time point in the diagnostic trajectory, irrespective of their age. To assess an increased brain atrophy rate due to disease would require a longitudinal study rather than this cross-sectional study. Potentially, data acquired in clinical routine could also be used to support such a longitudinal study design.

Finally, positively diagnosed dementia subjects showed significantly lower brain volume compared to negatively diagnosed subjects. This is in agreement with literature, in which it has been reported that dementia is associated with brain atrophy. The fact that the system is able to reproduce such results underlines the feasibility of the approach.

### Limitations

There are a number of limitations to our study. First, inherently with routine clinical data there will be a selection bias, since patients undergo a scan after some indication. Second, the MS protocol contained primarily T2-weighted and PD-weighted images in contrast to the T1-weighted image in the other groups. Therefore, differences in image acquisition could have had impact on our results. In this light, results based on routinely acquired clinical data must always be interpreted with extra caution.

Thirdly, in this study we have looked at fairly global imaging biomarkers for the brain; ICV and brain volume. These can be robustly extracted from large portions of legacy imaging data. The feasibility of the system to support the extraction of more sophisticated quantitative imaging biomarkers remains to be shown.

### Future Perspectives

In our proof of principle study, we were able to reproduce some known associations from literature. This provides evidence that the system is potentially a useful research tool, e.g. in associating imaging biomarkers with other parameters such as gender, age, disease, or drug use.

In future work we will investigate whether the system can also be used to extract more sophisticated MR imaging biomarkers, including e.g. hippocampal volume, cortical thickness, and structural connectivity. Also, we aim to include more sophisticated natural language processing in the subject selection to eliminate the need of reviewing patient records for the clinical characterization. Our goal is to implement this system at other medical institutions to further increase the scale of which routinely acquired clinical imaging data is used for research. This also includes the linkage of imaging data with other routinely acquired health care information sources such as medical records in e.g. general practitioners databases, national vaccination databases or hospital databases. This enables us for example to conduct research on the effectiveness and safety of medications, vaccinations (Martínez-Sernández and Figueiras 2013) or treatments.

## Conclusion

In a proof of principle study we have shown that ICV and total brain volume can be extracted from legacy clinical imaging data, despite image heterogeneity. This IT infrastructure can be a useful tool for biomarker research on routinely acquired clinical imaging data at a hitherto unprecedented scale.

## Information Sharing Statement

The software described in this paper will be released as open source under the Lesser GNU Public License (LGPL) 3, except for the brain tissue segmentation method. Access can be requested by contacting the author (k.y.e.leung@erasmusmc.nl). The imaging data remains under the ownership of the Erasmus MC, University Medical Center Rotterdam, but the sharing of data is possible under collaborative efforts.
